# The Half-Size ABC Transporter FOLDED PETALS 2/ABCG13 Is Involved in Petal Elongation through Narrow Spaces in *Arabidopsis thaliana* Floral Buds

**DOI:** 10.3390/plants3030348

**Published:** 2014-08-15

**Authors:** Seiji Takeda, Akira Iwasaki, Kiyoshi Tatematsu, Kiyotaka Okada

**Affiliations:** 1 Graduate School of Life and Environmental Sciences, Kyoto Prefectural University, Sakyo-ku, Kyoto 606-8522, Japan; 2 Biotechnology Research Department, Kyoto Prefectural Agriculture Forestry and Fisheries Technology Center, Seika, Kyoto 619-0244, Japan; 3 Laboratory of Plant Organ Development, National Institute for Basic Biology, Okazaki, Aichi 444-8585, Japan; E-Mails: iwaaki0123@gmail.com (A.I.); ktatem@nibb.ac.jp (K.T.); kiyo@nins.jp (K.O.); 4 Department of Botany, Graduate School of Science, Kyoto University, Kyoto 606-8502, Japan

**Keywords:** *Arabidopsis thaliana*, floral organs, folded petals, petal elongation, ABC transporter, ABCG13

## Abstract

Flowers are vital for attracting pollinators to plants and in horticulture for humans. Petal morphogenesis is a central process of floral development. Petal development can be divided into three main processes: the establishment of organ identity in a concentric pattern, primordia initiation at fixed positions within a whorl, and morphogenesis, which includes petal elongation through the narrow spaces within the bud. Here, we show that the *FOLDED PETALS 2* (*FOP2*) gene, encoding a member of the half-size ATP binding cassette (ABC) transporter family ABCG13, is involved in straight elongation of petals in *Arabidopsis thaliana*. In *fop2* mutants, flowers open with folded petals, instead of straight-elongated ones found in the wild type. The epicuticular nanoridge structures are absent in many abaxial epidermal cells of *fop2* petals, and surgical or genetic generation of space in young *fop2* buds restores the straight elongation of petals, suggesting that the physical contact of sepals and petals causes the petal folding. Similar petal folding has been reported in the *fop1* mutant, and the petals of *fop2 fop1* double mutants resemble those of both the *fop1 and fop2* single mutants, although the epidermal structure and permeability of the petal surface is more affected in *fop2*. Our results suggest that synthesis and transport of cutin or wax in growing petals play an important role for their smooth elongation through the narrow spaces of floral buds.

## 1. Introduction

Petals have evolved to have various colors, shapes, and fragrances, and, thus, their morphogenesis is critical in attracting pollinators for plants and in horticulture for humans. Flowers usually develop three other distinct organs, each with a specialized function. In *Arabidopsis thaliana*, the floral primordia initiate at the flanks of the shoot apical meristem as floral meristems, subsequently sepals arise, first in the outermost concentric region, and cover the floral meristem, protecting the other inner organs as they develop. Gynoecia and stamens are reproductive organs: gynoecia arise in the center of the flower, surrounded by a number of stamens.

Petal development can be divided into three main processes. First is the identity determination, described by the floral ABCE model, which explains that the organ identity is established in a concentric pattern by combined functions of the floral homeotic genes [1,2,3,4]. In addition to these main factors, miRNA172 and other transcription factors such as *AINTEGUMENTA*, *LEUNIG*, *SEUSS*, *RABBIT EARS* (*RBE*), and *STERILE APETALA*, are involved in fine-tuning of expression of the floral ABCE genes in *Arabidopsis thaliana* [5,6,7,8,9,10,11,12,13]. The second process is primordia initiation at fixed positions within each concentric region or whorl. In many flowering plants, such as Brassicaceae, petals initiate at the inner site of the sepal boundary region, thus, the number of petals and sepals is the same. Usually, positions where floral organ primordia arise within a whorl are fixed relative to the adaxial-abaxial and lateral axes of floral meristem. Although the molecular mechanism for determination of initiation position remains unclear, some genes that are expressed in a position-dependent manner, such as *PETAL LOSS* (sepal boundary), *RBE* (petal primordia), *ROXY1* (young floral organ primordia), and *UNUSUAL FLORAL ORGANS* (which changes its expression pattern as a flower develops), seem to be involved in position-dependent primordia development [14,15,16,17,18]. The third process is petal morphogenesis after initiation, which is regulated by cell division, cell expansion, and endoreduplication, regulated by *JAGGED*, *BIGPETALp*, and *FRILL* genes [19,20,21,22,23,24,25,26].

Previously we showed a mutant, *folded petals 1* (*fop1*), which in *Arabidopsis thaliana* has a defect in the petal elongation process [27]. In the wild type, petals elongate straight through the narrow space between sepals and anthers in the floral bud. In *fop1*, petals become stuck in the bud during elongation, resulting in the formation of folded petals in the open flower. *FOP1* encodes a member of the WAX ESTER SYNTHASE/DIACYLGLYCEROL ACYLTRANSFERASE (WS/DGAT) family WSD11 and is expressed in growing petals, suggesting that FOP1 is involved in production of fatty acids that makes petal elongation smooth in narrow space in floral buds, and that petal morphogenesis is affected by the physical interaction of floral organs.

Here, we describe the characterization of another mutant, *folded petals 2* (*fop2*), which has a similar petal folding phenotype to *fop1*. Our results suggest that FOP2 regulates petal elongation at distinct stages of the same pathway of FOP1, and that export of cutin and/or wax to the surface of the petal epidermis is important for petal morphogenesis.

## 2. Results and Discussion

### 2.1. Folded Petals Phenotype in the Fop2 Mutant

**Figure 1:**
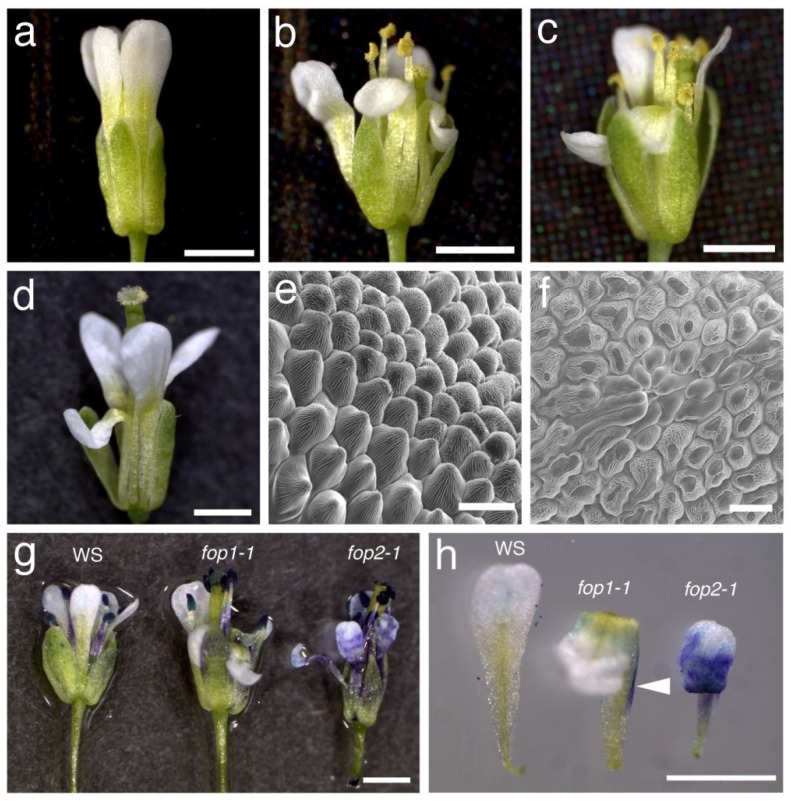
(**a**) Wild-type (WS) flower with straight petals; (**b**) *fop2-1* flower with folded petals; (**c**) *fop2-2*/SALK_046735 flower with folded petals; (**d**) *fop2-1 fop1-1* flower; (**e**,**f**) Scanning electron micrographs of wild-type (**e**) and *fop2-1* (**f**) petals; (**g**,**h**) Toluidine blue-stained flowers (**g**) and petals (**h**) of wild-type (WS), *fop1-1*, and *fop2-1*. Arrowhead indicates the edge staining of the *fop1-1* petal. Bars: a, b, c, d, g, h, 1 mm; e, f, 20 μm.

During a screen for floral organ defective mutants in *Arabidopsis thaliana*, we found a couple of mutants defective in the petal elongation process. Their flowers open with folded petals instead of wild-type straight-elongated ones, so we named these mutants *folded petals* (*fop*). One of these mutants, named *fop2-1*, produces flowers with folded petals with the shape of ‘N’ letter, instead of wild-type straight elongated petals (Figure 1a,b). About half of petals were unfolded (Table 1), but most of them were wrinkled, suggesting the trace of physical friction during petal elongation in flower buds (Supplementary Figure S1). After identification of the *FOP2* gene by map-based cloning (see below) we analyzed a SALK T-DNA insertion line (SALK_046735), which we named *fop2-2*, and found that this line showed the weaker but similar petal defects to *fop2-1* (Figure 1c and Figure S1). Since the *fop1* mutant showed similar petal defects [27], we examined the relationship between *fop1* and *fop2* by generating the double mutant. Flowers in the *fop2-1 fop1-1* double mutant showed similar petal folding to single mutants, and this phenotype was not enhanced in the double mutant as compared to the *fop2* single mutant (Figure 1d and Table 1), suggesting that *FOP1* and *FOP2* are involved in the same pathway of petal elongation.

To examine the petal defects in more detail, we analyzed the structure of the petal epidermis using scanning electron microscopy. In wild-type, petal epidermal cells are conical-shaped and epicuticular nanoridges cover their surface (Figure 1e). In *fop2-1*, many epidermal cells are flattened and lack the epicuticular nanoridges at their tip (Figure 1f). This defect was also observed on the *fop1* petal epidermis, suggesting that *fop2* petals make physical contact with sepals, as do the *fop1* petals [27]. In several regions on the *fop2-1* petal surface, a number of long cells lacking epicuticular nanoridges seemed to be aggregated at one site (Figure 1f). This phenotype was not detected in the wild type or *fop1*, suggesting that *FOP1* and *FOP2* have different roles in determining the petal epidermal character.

Some other mutants showing the petal-folding phenotype are sensitive to dye immersion due to the lowered repellency of petal surface [28,29,30]. We examined the surface repellency of *fop2* by immersing petals in toluidine blue solution [31]. Petals from wild-type flowers were not stained, but the *fop2* petals were stained in the blade region (Figure 1g,h). Interestingly, the *fop1* petals were not stained except for the claw edge (Figure 1g,h), probably because this region contacts to the sepal surface and thus has reduced repellency [27]. These results indicate that the petal repellency is lower in *fop2* than in *fop1*, suggesting that *FOP2* and *FOP1* have distinct functions.

Together, our results suggest that *FOP1* and *FOP2* regulate petal elongation in two distinct stages of the same pathway.

**Table 1:** Floral organ number of the *fop2-1* single and double mutants.

Genotype	Sepal	Petal		Stamen	Carpel
		Unfolded	Folded		
WS	4 ± 0	4 ± 0	0 ± 0	5.55 ± 0.60	2 ± 0
*fop2-1*	4 ± 0	2.15 ± 0.75	1.85 ± 0.75 ^a^	5.90 ± 0.45	2 ± 0
*fop2-1 fop1-1*	4 ± 0	1.85 ± 1.27	2.15 ± 1.27 ^a^	5.75 ± 0.44	2 ± 0
*prs-1 fop2-1*	4 ± 0	3.95 ± 0.22	0.55 ± 0.22	5.95 ± 0.22	2 ± 0

Twenty flowers were examined in each line. Numbers indicate the average organ number ± SD. ^a^ No significant difference each other (Student’s *t*-test, *p* > 0.05).

### 2.2. Surgical and Genetic Generation of Space in a Floral Bud Restored Petal Elongation

**Figure 2:**
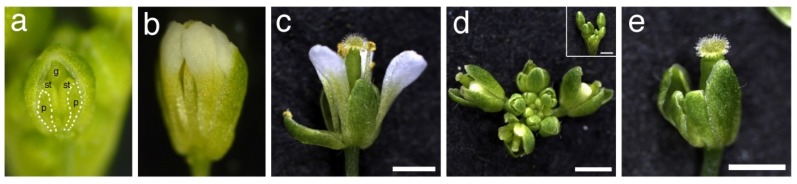
(**a**) *fop2-1* flower whose sepal is removed at stage 11. Petals are indicated by white dotted line. p, petal; st, stamen; g, gynoecium; (**b**) Same flower as (**a**) after three days. Note the straight elongation of petals; (**c**) *prs-1 fop2-1* flower; (**d**) Inflorescence of *prs-1 fop2-1*. Inset is inflorescence of *fop2-1*; (**e**) *ap3-5 fop2-1* flower. Bars: 1 mm.

We hypothesized that petal folding of *fop2* is caused by the narrow space in the floral buds, as shown in the *fop1* mutant [27]. To elucidate this, we removed the sepals from the *fop2-1* floral buds prior to flower opening (Figure 2a, around floral stage 10 to 12 as defined in [32]). After three days, we found that all examined flowers restored the straight elongation of petals (Figure 2b and Figure S2). We also examined the *fop1 fop2* double mutant in the same way, with similar results (Figure S2). Next, we investigated the *pressed flower-1 (prs-1) fop2-1* double mutant. *prs-1* is defective in the development of lateral domains of organs, and raises flowers with narrower sepals, resulting in “open” floral buds [33]. The floral buds of *prs-1 fop2-1* double mutants generate space in these open buds, compared to the closed buds in *fop2-1* (Figure 2d and inset), and almost all petals elongated straight in the double mutant (Figure 2c and Table 1). These results indicate that surgical or genetic generation of space in a floral bud restores the straight elongation of *fop2* petals. Taken together, the data suggest that the *fop2* lacks lubricant-like materials that allow petals to elongate smoothly in the narrow space in a floral bud.

We also investigated flowers of the *ap3-5 fop2-1* double mutant, whose petals were transformed to sepals, and found that the second whorl organ elongated normally (Figure 2e). This suggests the possibility that *FOP2* function is restricted to the organs with petal identity, and that sepals may have other ABCG transporters involved in cuticle wax deposition.

### 2.3. Mapping and Characterization of the FOP2 Gene

**Figure 3:**
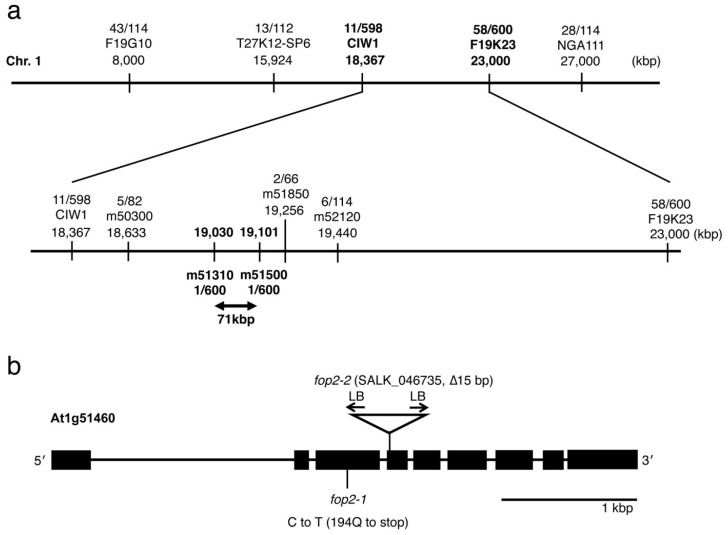
(**a**) Diagram of *FOP2* mapping. DNA markers and number of recombinants are indicated; (**b**) Genomic structure of the *FOP2* gene. Point mutation of *fop2-1* and T-DNA insertion, which causes the 15 bp deletion, in *fop2-2*/SALK_046735 are indicated. LB, left border of T-DNA. See Figure S2 for *FOP2* genomic sequence.

To characterize the molecular function of *FOP2*, we mapped the *FOP2* gene on chromosome 1 and identified a mutation in *At1g51460* (Figure 3). The *fop2-1* mutation is a nucleotide substitution of cytosine to thymine in the third exon, causing the change of a glutamine at the position of 194 to a premature stop in the amino acid residues (Figure 3b and Figure S3). We analyzed the SALK_046735 line, in which the T-DNA is inserted in the 4th exon of *At1g51460*. Sequencing of border regions revealed that the T-DNA caused a 15 bp deletion in the 4th exon (Figure 3b and Figure S3). This line showed the same petal-folding phenotype as shown above (Figure 1c).

*FOP2* encodes a member of the ATP-binding cassette (ABC) transporter family protein, ABCG13, which has been already reported by Panikashvili *et al.* [34]. They showed the folded-petal phenotype in SALK_046735 (*abcg13*) and RNAi-based knockdown lines, and several species of cutin monomers were reduced in these flowers. We examined the *FOP2* expression pattern by generating *FOP2* *genomic:* *GUS* translational fusion lines, and found that *FOP2* was expressed in petals and ovule integuments, as well as the L1 layer of peduncles (Figure S4). The expression pattern is largely consistent with *promoter:GUS* transcriptional fusion results from Panikashvili *et al.* [34].

### 2.4. Molecular Function of FOP2 in Petal Elongation Process

FOP2/ABCG13 belongs to the WHITE-BROWN COMPLEX (WBC) subfamily, members of which are half-transporter proteins [35,36]. ABCG11/AtWBC11/DSO and ABCG12/CER5 in the same subfamily function as homodimers or heterodimers on plasma membranes *in planta* [37], thus, FOP2/ABCG13 would interact with itself or the other WBC members on plasma membrane of petal epidermal cells. Our results from petal epidermis characterization, petal surface repellency tests and the phenotype of the double mutant suggest that production and deposition of the lubricant-like substrates, mediated by FOP1/WSD11 and FOP2/ABCG13, respectively, are a critical process for smooth elongation of petals in the narrow space in floral buds. Since the known half-size ABCG transporters are involved in secretion of both cutin and wax [34,38,39,40,41,42], it is possible that FOP2 transports the FOP1 products to the petal surface. Interestingly FOP1/WSD11 is located to the plasma membrane, suggesting that FOP2 and FOP1 may co-localize on the plasma membrane of petal epidermal cells. Identification of substrates and products of FOP1 and FOP2 will unveil the molecular mechanism of petal elongation in detail.

## 3. Experimental Section

### 3.1. Plant Lines and Growth Conditions

The Wassilewskija (WS), Columbia (Col), and Landsberg *erecta* (L*er*) ecotypes of *Arabidopsis thaliana* were used as wild-types. The *fop2-1* mutant was isolated from an M2 population of ethyl methanesulfonate-mutagenized *long hypocotyl 5* (*hy5*) in which the *HY5* genomic fragment was transformed [43]. *fop2-2/*SALK_046735, a T-DNA insertion mutant, was obtained from the SIGnAL Website [44] and the Arabidopsis Biological Resource Center [45]. Plants were grown on MS media or vermiculite in small pots under long-day conditions (16 h light and 8 h dark) with white light at 22 °C to 24 °C.

### 3.2. Histology and Microscopes

Images were captured using an S8AP0 binocular microscope or DM2500 light microscope equipped with an EC3 digital camera system (Leica, Wetzlar, Germany). For scanning microscopy, samples were directly observed with JSM-5800 (JEOL). For toluidine blue staining, flowers were immersed in TB solution (0.05% toluidine blue (w/v, Waldeck, Münster, Germany) and 0.01% Tween 20 (v/v)) for 5 min and washed in water. At least fifteen (WS and *fop2-1*) and five (*fop1-1*) flowers were examined, respectively.

### 3.3. Cloning and Expression Analysis of the FOP2 Gene

F2 plants generated by crossing the *fop2-1* with Col and L*er* were used for mapping. DNA marker information was obtained from The Arabidopsis Information Resource [46] and generated using polymorphisms between ecotypes. For genomic-GUS translational fusion, we fused the 5.7 kb genomic fragments including FOP2 5'-promoter and coding regions (without stop codon), β-glucuronidase (GUS), and 1.7 kb 3' region, and cloned into the pGWB1-NB1 binary vector, and transformed to plants by a vacuum infiltration procedure with the *Agrobacterium* strain ASE1.

## 4. Conclusions

Our results suggest that a half-size ABC transporter, FOP2/ABCG13, is involved in the transport of the possible lubricant materials on the developing petal surface. It is possible that FOP2/ABCG13 transports the products of FOP1/WSD11 on the plasma membrane of petal epidermis, resulting in the smooth elongation of petals through the narrow spaces in floral buds. Main future works would be (1) examining the molecular relation between FOP2 and FOP1, including spatiotemporal expression/localization analysis *in planta*, and enzymatic activity assay; and (2) identifying the substances that function as lubricants during petal elongation. Our results propose the possibility to control the petal morphogenesis by modification of enzymes or transporters, or by exogenous application of these products/substances.

## Supplementary Files

Supplementary File 1
