# Supplementary material for: The Half-Size ABC Transporter FOLDED PETALS 2/ABCG13 Is Involved in Petal Elongation through Narrow Spaces in Arabidopsis thaliana Floral Buds
Source: Plants (Basel). 2014 Aug 15;3(3):348–58. doi: 10.3390/plants3030348 (PMC4844351; doi:10.3390/plants3030348)
Supplement: Supplementary File 1 [file plants-03-00348-s001.pdf]

Supplementary Material

Figure S1. (**a**) WS petal. (**b**) Wrinkled and unfolded petal in *fop2-1*. Arrowheads indicate the wrinkled sites. Bars: 1 mm. (**c**) Petal character of WS, *fop2*, and *fop1* mutants. Examined flower number: WS, 20; *fop2-1*, 58; *fop2-2*, 38; *fop1-1*, 26.

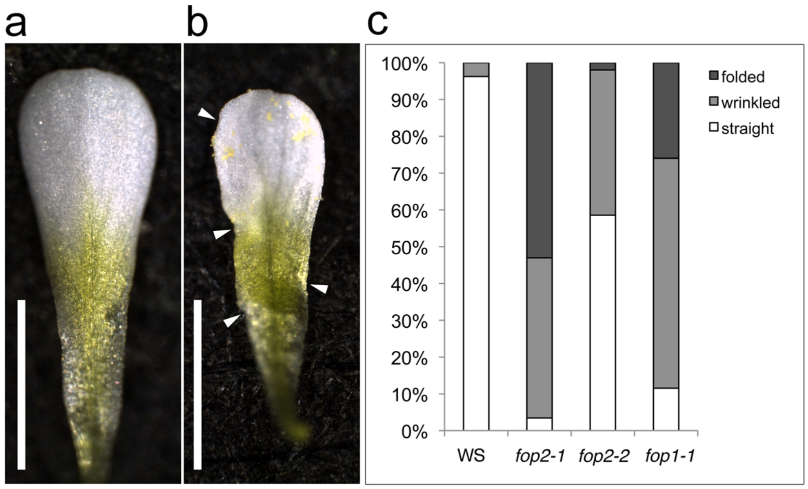

Figure S2. Sepal removal at early stage restores straight elongation of petals in *fop2-1* and *fop1-1 fop2-1* mutant flowers. Flowers with red square are with folded petals. *fop2-1*: n = 12, *fop1-1 fop2-1*: n = 16.

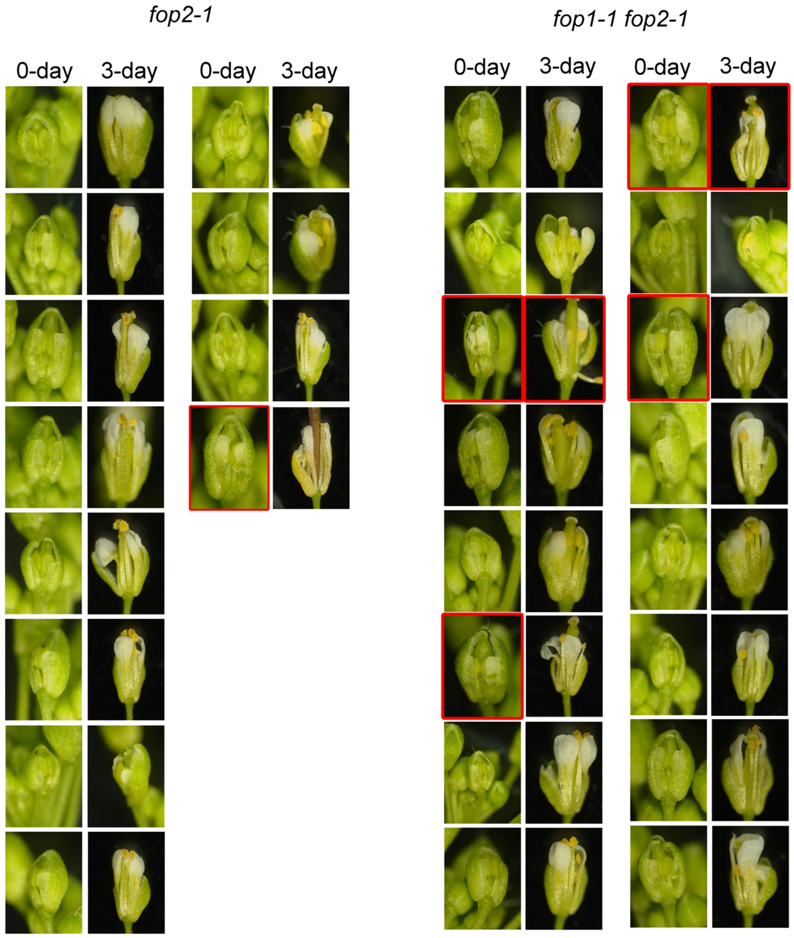

Figure S3. *FOP2/At1g51460* genomic sequence. Bold letters represent exons. Marked nucleotides by red and blue indicate the point mutation in *fop2-1* (changed to T) and 15 bp deletion caused by T-DNA insertion in *fop2-2*/SALK_046735, respectively.

acacttttctctcccaaagaaacaaaaaaaaaaactcttaagctagaacaagtttaaaactcttcgctcagttttcgatctatct
aacaaaagcatac**ATGACTACGCCGGAGGGGGCGATGTATGTGGCGTGGGAGGATCTTACGGTGGTGATACCAAACTTCGGTGAA**
GGAGCAACTAAGAGATTACTGAATGGAGTGAATGGTTGTGGTGAGCCAAATAGGATCTTAGCTATTATGGGTCCTTCAGGTTCAG
GCAAATCTACGCTTCTTGATGCTTTAGCAGgtcccttctctctctcagacacacacacccatacatgtcaagattagctcgtc
gtttttattatgtatttcttttattgttaaatggttttcctctattatataaatctcaaataagctgaagcaacatcgaccaaca
attaaaagatatagtttttagaggaatttaacaaccaaccacatttagctttgaaaccaacattaaaggatgagacttacccact
ttgaacctagtatttagtgaatcgttctatgtactctatatatatatatatataactataattaacaatatacatgacgttatgt
ctttcttttaaaaaattgtccaaggaaactttctcaatatactatgtaaaaagtgtttcacttttggaaaataagaaatttctgt
tttcttacattaaaaaatattcacaaatcttttagtaattttttatttggaattgataaggatttctgccattcgtagttaaattt
gagaatataagttagaatttatttgttagatatatatatatatatatatatataggtgtatgtctgtgcgtgggtgggtgtgtgt
cctattcaacataaaatatttaattattattaacctgtaaattcgtatgtaatcaaaattaactaatatgatacttgattagttt
tttgtatattggaaaaatacattactacaataaaattgataactttcctatttgaaagcaacaaactatgtacatactgtatcag
tatcacaatttggtgaaatgaaaatattaactgaagtttttgtactcttctgtaattttttttttttttcttctgtaactttgac
caaacgtaacatatatatatgtaaaagaaatttgagagcatgatataacatatttatgtatcaagatatttataactattgaaaa
acagtgctatatttatatctaagtttcatgtacacgatgagacatatatagatatttctaactttatatttatggattctagcta
ccaattttatttttaagtatagctatatactatcggagtactaaataaatattaaaaaaaaaatagttgcaattttcatgttct
taattaataaatttacatcgtcgtacatttagtattgaataaattgatagttacgtttctctaacgtagactatgtacagaaatt
ttgcagtaacatttcttattaaatcatattttaaatgttaaccaactaccaatcaccaatattctgacacaaatatttaggattg
cacgatcgtatcttcataccgacttgcgatctcttcacaagttgaacccggtttggtgactggtcggactctttgagggatgtta
cgttcaatccaaaaacaatccaaatcgattattattttcttgaccaaacctaaatttgattggattaagaagatctttattttat
gcttatgactaatcgaaaccaatttaattttactgtggctaatattcttgtaaaagcgaaaaacccaaaaccgggttataagatt
gttctatcaatgctataagattgttcaaaaacatgaaagatttaacatctaacattaaaaggtaaatttggtgatggtacag**GAA**
GATTAGCAGGGAATGTTGTAATGAGTGGCAAAGTACTTGTCAATGGCAAGAAGAGAAGACTTGACTTTGGTGCTGCCgtaagaaa
atttatcttttctctttagtttttacccttttacacaatcaaactattattattcatgaatgtcctaaactctcag**GCTTATGTG**
ACGCAAGAAGATGTGTTGCTAGGAACTTTGACAGTGAGAGAATCCATATCTTACTCGGCTCATCTCAGGCTCCCTTCAAAGCTTA
CCAGAGAAGAGATCAGTGACATTGTGGAAGCTACAATCACTGACATGGGTCTTGAAGAATGTTCAGACAGGACCATTGGAAACTG
GCATTTGCGTGGAATAAGCGGAGGAGAGAAGAAACGGCTTAGTATTGCCCTCGAGGTCTTAACAAAACCAAGTCTCCTCTTTCTA
GACGAACCAACTAGTGGACTGGACAGTGCTTCAGCTTTCTTCGTGGTTCAGATTCTTAGAAACATAGCAAGCAGTGGCAAAACTG
TGGTTTCTTCGATCCATCAGCCGAGTGGTGAGGTTTTCGCCCTCTTTGATGACCTGCTACTTCTGTCTGGAGGAGAAACTGTTTA
CTTTGGCGAAGCAGAATCTGCAACAAAGgtaataagatagagcattggcaagagtatataaccattatgctcatttgtgttgttg
ttgcttcgacattgcag**TTCTTTGGTGAAGCAGGTTTTCCTTGTCCAAGTAGACGGAATCCATCAGACCACTTCCTTCGCTGTGT**
CAATTCCGATTTCGATAATGTCACAGCGGCTTTGGTTGAATCTCGGAGAATCAACgtttgttttcctctgcccttcttgtttttt
catgaacttacaatactctcaagcttttgacctcgaccttttacatctgcag**GATTCATCTTTCTCTCTTCACCAACTACATGAA**
ACTACAAACACATTAGATCCTCTGGATGATATACCAACTGCAGAAATTAGAACAACACTTGTCAGAAAATTTAAGTGTTCACTTT
ATGCAGCAGCTTCAAGAGCAAGAATTCAAGAAATAGCATCGATAgtaagttacggttactacttcccttaaaactacaatgtatc
acataatgagagtctcataagcacagttataaaccttgtgcaacag**GTGGGGATTGTCACAGAAAGGAAAAAGGGGAGCCAAACT**
AATTGGTGGAAACAACTCAGAATACTTACTCAGCGATCTTTCATCAACATGAGTAGAGACTTGGGGTACTACTGGATGCGGATTG
CAGTCTACATAGTGTTATCCATTTGTGTCGGGTCAATCTTCTTCAACGTCGGGAGAAACCACACAAATGTTATGAGTACTGCAGC
TTGTGGCGGATTTATGGCAGGCTTTATGACATTCATGTCAATAGGAGGATTCCAATCCTTCATTGAAGAAATGAAGgtaaagagc
taaacagagaaaaattctctttcttcacctaaaaacttatcttatgttttagcatctttattcctgaaaacag**GTGTTTTCTCGC**
GAAAGGCTCAATGGACACTATGGGGTTGCAGTATACACTGTGTCTAATTTACTCTCCTCATTACCTTTCATAATCCTTATGTGCC
TCTCCACCAGCTCAATCACTATCTACATGGTGAGGTTCCAATCCGGAGGTTCTCATTTCTTCTACAACTGTCTCGACCTTATCTG
CGCGATTACAACTGTGGAGAGTTGCATGATGATGATAGCTTCAGTAGTCCCTAACTTCTTGATGGGAGTCATGTTGGGAGCTGGT
TACATTgtaagagtggcctctgtcgctctttcacctacaagctttcttatatctccaaaaactattctaaattgaaaaccgcttt
atccttcatctcatag**GGAATTATGGTGTTAAGTGCGGGGTTTTTCCGATTCTTCCCCGACTTACCAATGGTGTTCTGGCGATAC**
CCGGTGTCCTACATAAACTATGGTGCATGGGCACTACAGgtaaccaggttttctcaactttaagatgcagactatatcgtcaagt
agctatcttaaatgagcatacctgataaattgcag**GGAGCATACAAGAATGAGATGATTGGGGTGGAGTATGATTCTCCGTTACC**
CTTGGTACCAAAAATGAAAGGAGAACTCATTCTTCAAACTGTTCTAGGCATAAATCCAGAAAGTTCAAAGTGGTTGGATCTAGCA
GTTGTGATGATGATTCTCATTGGGTATAGGATTGCCTTCTTCGCCATCCTCAAGTTCCGGGAAAAGGTTTTCCCAGTTATTCACA
TGTTATACACAAAGAGAACTCTGAGCCATATCCAGAAAAGGCCTTCTTTCAGAAGAATGACACCTTTCCCTTCCAGGCGATACCC
TGTTCACCATGCTCTCTCTTCTCAGGAAGGACTTAACTCTCCACTGCATTAGaagcatatacagcaggatgcaatttccatatat
ttcaatatgtcagatgtcactctcttcaaacttgataaactgtttgtagcccgttgtgtaaggaaaaaaaaaagaaaagagagat
actatcattctgactaatgagatttgtttagc

Figure S4. GUS staining in *FOP2g:GUS* flowers. (**a**) GUS stains in petals, ovules, and L1 cells of peduncles. (**b**) Higher magnification of ovules in (**a**), showing the expression in outer and inner integuments (arrowheads). n, nucellus; ii, inner integuments; oi, outer integumetns. Bars: 20 µm.

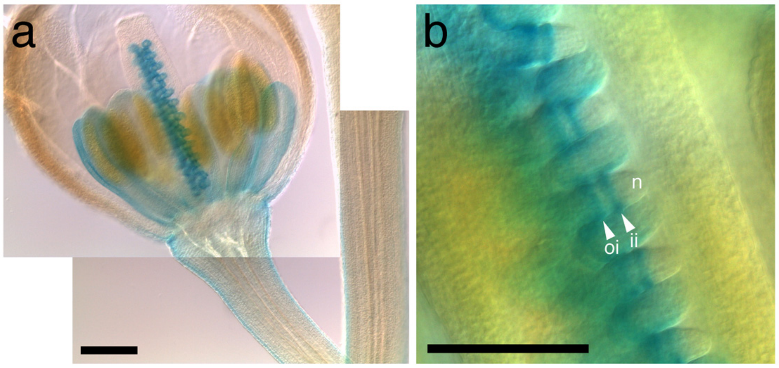

© 2014 by the authors; licensee MDPI, Basel, Switzerland. This article is an open access article distributed under the terms and conditions of the Creative Commons Attribution license (http://creativecommons.org/licenses/by/3.0/).
